# Pyogenic granuloma as a surrogate indicator of deep seated foreign bodies: a case report

**DOI:** 10.4076/1757-1626-2-7354

**Published:** 2009-08-05

**Authors:** Ernest A Azzopardi, Christian Borg Xuereb, Srinivasan Iyer

**Affiliations:** 1 Department of Plastic Surgery, Wexham Park HospitalWrexham Road, Slough, SL2 4GQ BerkshireUK; 2 Department of Health Psychology, Aston UniversityBirminghamUK; 3 Department of Plastic Surgery, Wexham Park HospitalWexham Road, Slough, SL2 4GQ BerkshireUK

## Abstract

Although pyogenic granulomas are often clinically associated with foreign bodies or recurrent traumatic injury, this association is not well documented. We report a case of a recurrent, intractable pyogenic granuloma due to a missed foreign body. An extensive search retrieved no previous literature reporting this association. This lack of evidence bases may hinder the hand surgeon from extending the wound incision and thorough exploration. Recurrent pyogenic granulomas should lead the hand surgeon to entertain the possibility of a missed foreign body.

## Case presentation

A seven year old right-hand dominant previously well Caucasian male presented with palmar pain and discharge three days after a fall on his outstretched right palm. On examination the patient was unable to flex his right index finger (RIF). Plain x-ray was normal. In theater a 1 cm long wooden splinter was delivered through a puncture wound in the palm, from the subcutaneous tissue overlying the RIF. Over the next seven weeks a persistent granuloma developed. This was unresponsive to silver nitrate cautery, hydrocortisone cream, and surgical excision and diathermy. Seven weeks later a wooden splinter was delivered through the granuloma, from the soft tissue overlying the second metacarpal. Following intensive physiotherapy and intravenous antibiotics, the patient made an uneventful recovery.

## Discussion

No studies were retrieved linking recurrent pyogenic granulomata to the presence of foreign bodies from an electronic, multidisciplinary search across the main databases. However, four case reports documented the formation of a pyogenic granuloma following silicon punctual plugs [1-4]. Northington and Huang (2004) reported the formation of a pyogenic granuloma due to an exposed sternal wire [5]. The aetiology of pyogenic granulomata is as yet unknown, although trauma, infection and preceding dermatoses have all been suggested [6]. In our patient, the foreign body was identified deep in the subcutaneous tissue of the hand, which was dressed in a bulky bandage reducing the chance of friction causing the granuloma. Based on our observations we speculate that the occurrence of a recurrent pyogenic granuloma over the site of previous trauma, which is resistant to treatment should alert the hand surgeon towards suspicion of an underlying foreign body.

## Conclusion

Although pyogenic granulomas are anecdotally associated to recurrent trauma or foreign body reactions, this association is not documented in the literature. This case report illustrates the necessity of the hand surgeon encountering a persistent and intractable pyogenic granuloma, to entertain this association.

## Abbreviation

RIF: Right index finger
